# *CACNA1C* hypermethylation is associated with bipolar disorder

**DOI:** 10.1038/tp.2016.99

**Published:** 2016-06-07

**Authors:** A Starnawska, D Demontis, A Pen, A Hedemand, A L Nielsen, N H Staunstrup, J Grove, T D Als, A Jarram, N L O'Brien, O Mors, A McQuillin, A D Børglum, M Nyegaard

**Affiliations:** 1 Department of Biomedicine, Aarhus University, Aarhus, Denmark; 2 The Lundbeck Foundation Initiative for Integrative Psychiatric Research, iPSYCH, Denmark; 3 Center for Integrative Sequencing, iSEQ, Aarhus University, Aarhus, Denmark; 4 Department of Molecular Biology and Genetics, Aarhus University, Aarhus, Denmark; 5 Molecular Psychiatry Laboratory, Division of Psychiatry, University College London, London, UK; 6 Research Department P, Aarhus University Hospital, Risskov, Denmark

## Abstract

The *CACNA1C* gene, encoding a subunit of the L-type voltage-gated calcium channel is one of the best-supported susceptibility genes for bipolar disorder (BD). Genome-wide association studies have identified a cluster of non-coding single-nucleotide polymorphisms (SNPs) in intron 3 to be highly associated with BD and schizophrenia. The mechanism by which these SNPs confer risk of BD appears to be through an altered regulation of *CACNA1C* expression. The role of *CACNA1C* DNA methylation in BD has not yet been addressed. The aim of this study was to investigate if *CACNA1C* DNA methylation is altered in BD. First, the methylation status of five CpG islands (CGIs) across *CACNA1C* in blood from BD subjects (*n*=40) and healthy controls (*n*=38) was determined. Four islands were almost completely methylated or completely unmethylated, while one island (CGI 3) in intron 3 displayed intermediate methylation levels. In the main analysis, the methylation status of CGI 3 was analyzed in a larger sample of BD subjects (*n*=582) and control individuals (*n*=319). Out of six CpG sites that were investigated, five sites showed significant hypermethylation in cases (lowest *P*=1.16 × 10^−7^ for CpG35). Nearby SNPs were found to influence the methylation level, and we identified rs2238056 in intron 3 as the strongest methylation quantitative trait locus (*P*=2.6 × 10^−7^) for CpG35. In addition, we found an increased methylation in females, and no difference between bipolar I and II. In conclusion, we find that *CACNA1C* methylation is associated with BD and suggest that the regulatory effect of the non-coding risk variants involves a shift in DNA methylation.

## Introduction

Bipolar disorder (BD) is a common complex mental disorder characterized by episodes of mania, hypomania and depression. The disorder affects ~1% of the population and the genetic risk component is high with heritability estimates reaching 89%.^1, 2^ *CACNA1C* is one of the most consistently associated BD genes. *CACNA1C,* encodes the pore-forming α1C subunit of the L-type voltage-gated calcium channel also referred to as Ca_v_1.2 α1C subunit. In the central nervous system, Ca_v_1.2 channels are predominantly located in the postsynaptic dendritic processes and somata. On membrane depolarization, the Ca_v_1.2 channels allow postsynaptic influx of calcium ions coupled with activation of neuronal transcription factors involved in dendritic development, neuronal survival, synaptic plasticity, as well as memory formation and learning.^3, 4^

The first genome-wide association study (GWAS) that suggested *CACNA1C* to be a susceptibility gene for BD was performed on 1461 BD patients and 2008 control individuals, collected as part of the Systematic Treatment Enhancement Program for Bipolar Disorder (STEP-BD) and the University College London (UCL) sample. This GWAS showed nominally significant association of rs1006737 located in intron 3 of *CACNA1C* (*P*=1 × 10^−4^) with BD.^5^ Subsequently, a larger GWAS with 4387 BD cases and 6209 controls, including overlapping samples from the first study, further supported the association of rs1006737, now reaching borderline genome-wide significance (7 × 10^−8^).^6^ A meta analysis of BD carried out by the Psychiatric Genomic Consortium (PGC) Working Bipolar Group, encompassing 11 974 BD cases and 51 792 controls, identified a nearby single-nucleotide polymorphism (SNP), rs4765913, as the most significant risk SNP in *CACNA1C* (*P*=1.52 × 10^−8^).^7^ The two SNPs are in moderate linkage disequilibrium (LD) (*r*^2^=0.44) and both are positioned within what is today reckoned as a narrow intronic risk locus of *CACNA1C*. The most recent published GWAS of BD, performed in 9747 BD patients and 14 278 controls, also found association of rs4765913 with BD (*P*=9.96 × 10^−6^).^8^

Shortly after the first GWAS implicated rs1006737 in BD, several studies found association of the same SNP with schizophrenia (SZ).^9, 10, 11^ A large GWAS of SZ (including 36 989 cases and 113 075 controls) performed by the PGC identified a highly significant association of rs2007044 in intron 3 of *CACNA1C* with SZ (*P*=2.63 × 10^−17^).^12^ A recent meta analysis further supported association of *CACNA1C* with SZ, with rs4765905 positioned in intron 3 as the best finding (*P*=5.14 × 10^−17^).^13^ It is clear, that although arrays, imputation methods and the best-associated SNPs may vary between studies, the association with both BP and SZ robustly originates from a relatively narrow set of SNPs positioned in the large intron 3 of *CACNA1C*.

In healthy subjects, the *CACNA1C* risk allele (A-allele) at rs1006737 has been associated with increased hippocampal activity during emotional processing and increased prefrontal activity during executive cognition, specific patterns of brain activity that are associated with mental illness.^9^ In addition, the risk allele have also been associated with higher psychopathology scores for depression and anxiety,^14^ higher paranoid ideation scores,^15^ increased activity in right amygdala during fear-face recognition,^16^ and increased amygdala volume,^17^ confirming the important role of this risk locus in the etiology of psychiatric traits.

As the risk SNPs in *CACNA1C* do not lead to changes in the coding sequence of the protein, the central hypothesis guiding the current research effort is that intron 3 contains important regulatory functions. Results from a recent study suggest that the intron 3 risk SNPs have a regulatory effect on three-dimensional genome architecture associated with chromosomal loopings and thus influence *CACNA1C* expression through interactions with its transcription start site.^18^ DNA methylation is a powerful key regulator of gene expression across time, tissue and lifespan,^19, 20^ and because it is well-established that DNA methylation levels can be influenced by nearby genetic variants, methylation changes can provide a functional mechanism by which non-coding sequence variation can result in phenotypic variability.^21^

In this study, we investigated if DNA methylation of *CACNA1C* is altered in BD cases. A systematic study of all five CpG Islands (CGIs) across the gene was first performed to get an overview of its methylation landscape. Subsequently, the DNA methylation of a CGI positioned in intron 3 of *CACNA1C* was analyzed in a larger case–control sample revealing significant hypermethylation in BD cases, which was partly driven by genotypes in the established intronic risk locus.

## Materials and methods

### Subjects and sample preparation

BD subjects and control individuals were collected at UCL and collaborating clinical centers. The UCL cases comprised Caucasian individuals who were ascertained and received clinical diagnoses of BD according to UK National Health Service (NHS) psychiatrists at interview using the categories of the International Classification of Disease version 10 (ICD10), as described in a previous BD GWAS.^5^ The UCL control subjects were recruited from London branches of the National Blood Service, from local NHS family doctor clinics and from university student volunteers also as described before.^5^ All control subjects were interviewed with the Schizophrenia and Affective Disorders Schedule-Life Time version to exclude all psychiatric disorders.^22^ The sample is partly overlapping with the UCL sample described in Sklar *et al.*^5^ and the PGC mega-analysis of BD.^7^ All subjects from the UCL sample, where DNA was still available and where the DNA was extracted from whole blood, were used for the study, in total 725 subjects. In addition, 176 new cases and controls were included, collected using the same inclusion criteria, reaching a final sample size of 901 subjects. For a description of the overlap see Supplementary Table 1.

For the UCL BD cases, pedigree and family history data were collected based on self-reports by BD participants. The BD cases were categorized according to whether there was a history of mental illness, suicide or alcohol dependence from only the maternal or paternal side of the family.

The study was approved by the National Research Ethical Committee and all participants provided written informed consent.

Genomic DNA was extracted from frozen whole blood using standard procedures. For methylation analysis, 200 ng genomic DNA from each individuals was bisulfite converted with the EZ DNA Methylation Kit (Zymo Research, Irvine, CA, USA) according to manufacturer's instructions.

### EpiTYPER

EpiTYPER assays were designed for a total of 169 CpG sites spread over 5 CGIs using the EpiDesigner software (Sequenom, San Diego, CA, USA; www.EpiDesigner.com). After PCR amplification of bisulfite-converted DNA, the amplicons were treated with Shrimp Alkaline Phosphatase (Sequenom) followed by *in vitro* transcription. The resulting product was treated with RNase A to cleave the RNA at each U nucleotide using the T-Cleavage MassCLEAVE Kit (Sequenom). The fragments were then conditioned with Clean Resin (Sequenom) with the protocol provided in the kit and dispensed onto a SpectroCHIPII (Sequenom) and then analyzed on a MassARRAY Workstation in EpiTYPER mode. Quality control and initial data analysis were performed with the use of EpiTYPER (version 1.2, Sequenom). Oligonucleotide sequences for the EpiTYPER assays are provided in Supplementary Table 2.

### iPLEX

iPLEX assays for six selected CpG sites in CGI 3, located in intron 3 of *CACNA1C*, were designed using Assay Designer in Typer 4.0 (Sequenom) using bioinformatically bisulfite-converted sequences of the targeted region. Inosine was added to oligonucleotide sequences that contained overlapping CpGs or SNPs. No more than one inosine was allowed in an oligonucleotide to ensure binding specificity. The oligonucleotide sequences for the iPLEX assays are listed in Supplementary Table 3. The amplified products were prepared according to iPLEX manual and dispensed on the same equipment as described above. The data were analyzed in Allelotype mode. Quality control and detection of allele ratios (as a measure of methylation status) were performed using Typer 4.0 (Sequenom). The data were subsequently imported into R (www.r-project.org) for statistical analyses. During all experimental work and QC, the investigator was blinded to affection status. Cases and controls were in addition spread in between each other on each plate during bisulfite conversion and Sequenom analysis to avoid experimental batch effects.

### Statistical analyses

A non-parametric Mann–Whitney test was used to compare the levels of DNA methylation between cases and controls, males and females, cases with bipolar I and bipolar II, and cases with a positive versus a negative familial history. For the analysis of familial history, cases with a maternal history of psychiatric disorder (maternal) were compared with cases with no mental disorder in the family (none). A similar analysis was carried out comparing cases with paternal history versus none. Methylation quantitative trait loci (mQTL) analysis was performed using the 725 subjects from the UCL sample with Affymetrix 500 K Array genotypes available through the NIMH Repository (https://www.nimhgenetics.org/available_data/data_biosamples/gwas_data.php).

The mQTL analysis was performed using linear regression in PLINK^23^ including genotyped SNPs located in a region that encompassed the *CACNA1C* gene plus 5 kb up- and downstream (focused analysis) or all genotyped SNPs on chromosome 12 (full analysis). For the most significant CpG site in CGI 3, a beta regression analysis was performed to assess the contribution of case–control status, genotype and gender on DNA methylation level. A genetic association analysis in the UCL sample was performed for 109 genotyped SNPs in *CACNA1C* and a 5 kb up- and downstream region using a linear regression in PLINK and an additive model.

## Results

### Association of CACNA1C methylation with BD

**Figure 1:**
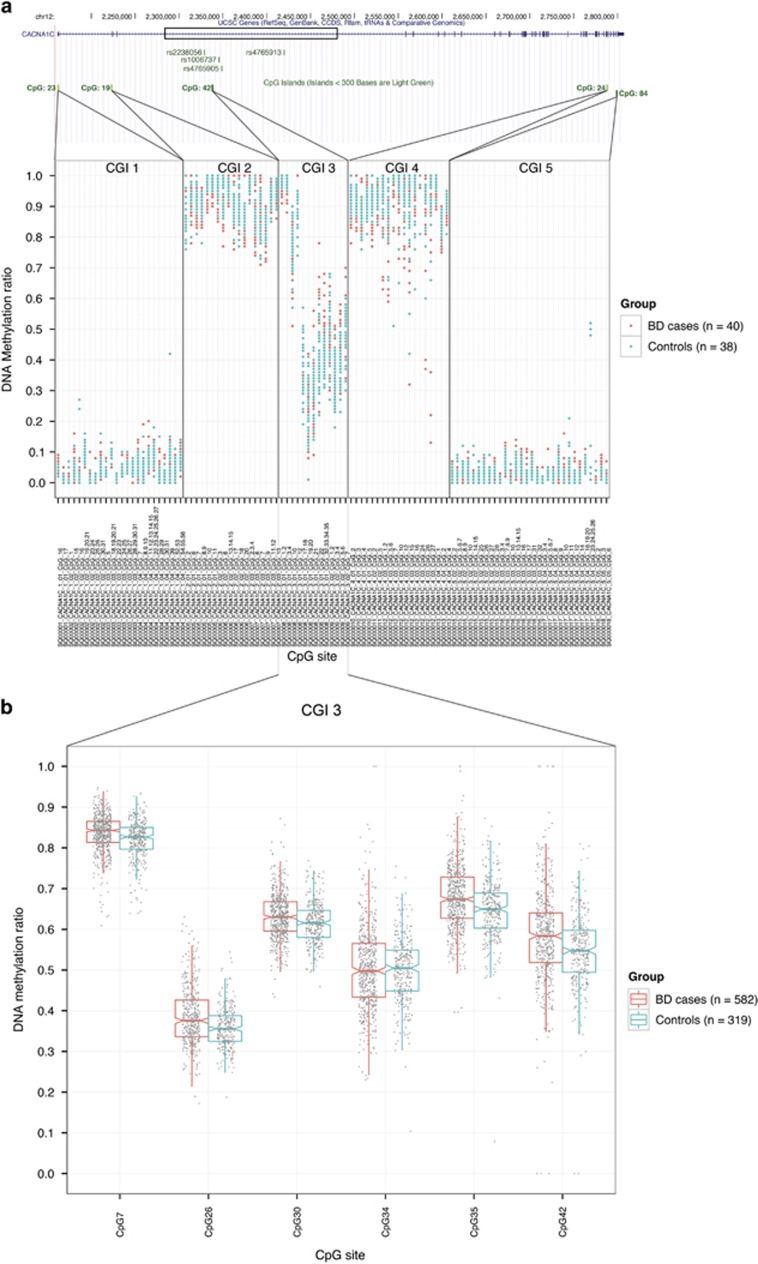
DNA methylation of *CACNA1C* in BD cases and controls. (**a**) Shows the gene structure of *CACNA1C* and methylation landscape across its all five CpG islands in BD cases and controls; (**b**) shows detailed analysis of DNA methylation levels of CpG island 3 in *CACNA1C* in BD cases in comparison to controls. BD, bipolar disorder. CGI, CpG island.

The initial methylation profile of the five *CACNA1C* CGIs in DNA derived from the blood of BD subjects (*n*=40) and controls (*n*=38) using EpiTYPER identified a large difference in DNA methylation between the islands with relatively little overall difference between individuals. In general, CGI 1 and CGI 5 were found to have low methylation levels, CGI 2 and CGI 4 were highly methylated, while CGI 3 displayed intermediate methylation (Figure 1a).

The main methylation analysis of CGI 3 included six CpG sites that were evaluated in BD subjects (*n*=582) and control individuals (*n*=319). From this analysis, a relatively small but highly significant degree of hypermethylation in BD subjects was identified for five of the six CpG sites (Figure 1b and Table 1). The most significant difference in methylation between BD subjects and controls was found for CpG35 (*P*=1.16 × 10^−7^, Δ*β*=0.03) (Figure 1b and Table 1). The DNA methylation levels were highly correlated between the six sites (Supplementary Table 4).

### Identification of mQTL

Because it is well-known that genetic variation can influence methylation levels^24, 25^ we speculated whether methylation at CGI 3 was influenced by nearby SNPs. A correlation analysis including methylation levels at the six CpG sites and SNPs on chromosome 12 was performed for all 725 individuals for whom genotype information was available (Supplementary Table 1). This analysis was performed by including all genotyped SNPs within *CACNA1C*, as well as 5 kb upstream and downstream of the gene (109 SNPs, 24 of whom were independent). This analysis will henceforth be referred to as the focused analysis. In addition, an analysis including all SNPs on chromosome 12 (15 882 SNPs, 2634 independent) was performed, referred to as the full analysis.

**Figure 2:**
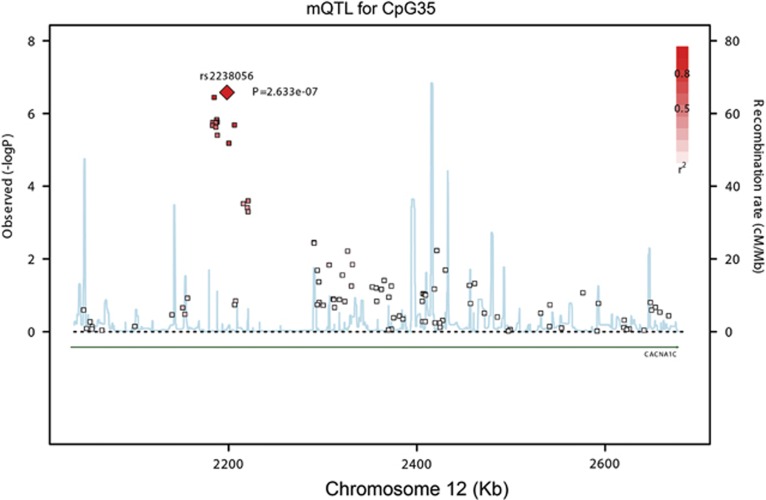
Overview for mQTL analysis between all SNPs genotyped in *CACNA1C* in the UCL sample and DNA methylation levels at the most significantly hypermethylated CpG site (CpG35). mQTL, methylation quantitative trait loci; SNP, single-nucleotide polymorphism; UCL, University College London.

In the focused analysis, we found multiple SNPs that were acting as significant mQTL for sites in CGI 3 (Table 1). All of these SNPs were located within the relatively narrow risk locus in intron 3. SNP rs2238056 was identified as the strongest mQTL (*P*=2.6 × 10^−7^) for CpG35, the most significantly associated CpG site in BD subjects (Figure 2). In the full analysis, all the SNPs that were identified as mQTL in the focused analysis were re-identified as the best mQTL SNPs, with exception of CpG26, where rs2239030 was replaced with rs4026015 as the most associated mQTL SNP (Table 1). Overall the analyses demonstrate that CGI 3 methylation is influenced primarily by SNPs within the intron 3 risk locus of *CACNA1C*. The mQTL for CpG35 remained significant after Bonferroni correction in both the focused and the full mQTL analyses (Table 1). Interestingly, the mQTL SNPs identified in this study were in moderate LD with the bipolar GWAS top hit rs4765913 (index SNP) with *r*^2^=0.32 for rs2239030, *r*^2^=0.3 for rs2238056 and *r*^2^=0.4 for rs10848634 according to results from the PGC Bipolar Disorder Working Group.^7^ This finding supports the hypothesis that the well-established risk SNPs in *CACNA1C* for BD may have a role in DNA methylation status.

**Figure 3:**
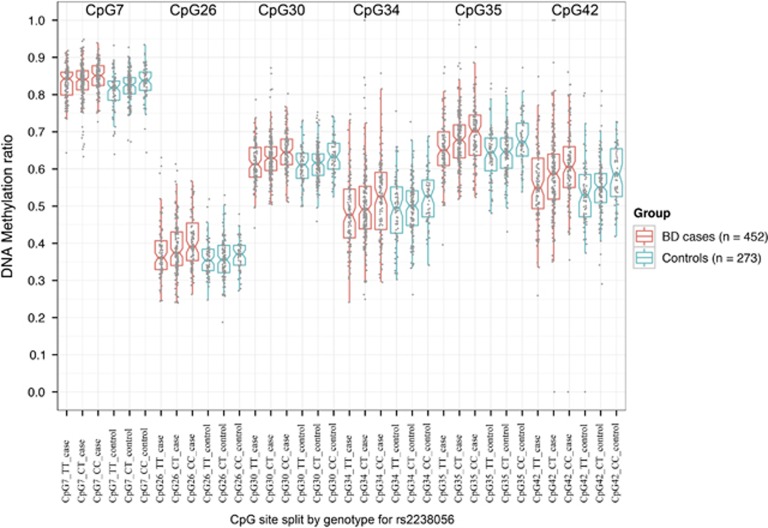
DNA methylation levels at six CpG sites in CpG island 3 of *CACNA1C* split by genotype of rs2238056, identified as most significant mQTL for the most significantly hypermethylated CpG site (CpG35). BD, bipolar disorder; mQTL, methylation quantitative trait loci.

For the best CGI 3 mQTL in our study (rs2238056), the minor C-allele was associated with increased DNA methylation levels for all of the examined CpG sites in CGI 3 (Figure 3). A similar analysis for rs1006737 showed that the risk minor A-allele was associated with increased DNA methylation for all CGI 3 CpG sites in a dose-dependent manner, which is consistent with these two minor alleles being in LD. This effect was found in both cases and controls (Table 1 and Supplementary Figure 1).

To test if the identified top mQTL could be risk SNPs themselves, we carried out a genetic association analysis in the UCL sample, including rs1006737 for comparison. Two of these top mQTL and rs1006737 were borderline significantly associated with BD in the UCL sample, with better *P*-values in the PGC-BP data set, most likely because of larger sample size (Supplementary Table 5).^26^ Interestingly, the mQTL rs2238056 is highly associated with SZ in the PGC SCZ 52 study (*P*=1.45 × 10^−13^).^12^ The data suggest that the mQTL may be risk SNPs themselves, although the high degree of LD in the region makes the identification of the true causal risk SNP(s) complex.

### Effect of gender, bipolar I/II and familial history

We finally investigated if any of the other known variables examined in the BD cases (Supplementary Table 1) could explain the variation in methylation among cases and controls. We found a hypermethylation in females compared with males. A gender-specific analysis confirmed a significant hypermethylation in cases both among females and males (Table 1). A beta regression analysis of CpG35 showed significant correlation of DNA methylation level with affection status (*P*=1.61 × 10^−5^), gender (*P*=2.27 × 10^−5^) and genotype of rs2238056 (*P*=6.10 × 10^−5^). All these covariates influenced methylation level to approximately the same extent. Odds of methylation increased by 15% for being a case, 15% for being a female and by 9% for each copy of the minor allele of rs2238056. No difference was found between cases with or without a parental history of psychiatric disease (*P*>0.05). Also, no difference was found between bipolar I or bipolar II patients (*P*>0.05); however, due to a small number of individuals with BD II diagnosis these results may not be generalizable. We conclude that the CGI 3 hypermethylation in cases is driven partly, but not entirely, by genotypes and by gender.

## Discussion

To our knowledge, this is the first study to investigate DNA methylation of *CACNA1C* in context of BD and with BD-associated genetic risk variants. We profiled the DNA methylation landscape of five CGIs in *CACNA1C* in blood-derived DNA and found CGI 3 to be significantly hypermethylated in BD subjects compared with controls. We also found that methylation was influenced by gender as well as by nearby genotypes.

Because of LD in the human genome, it is in general difficult to pinpoint the causal variation in a locus identified in a GWAS. For complex traits specifically it may be impossible to demonstrate that an implicated SNP is the causal variant. However, as pointed out in an editorial comment in Nature Genetics,^27^ there should at least be evidence that the variants identified in a GWAS show a consistent correlation with gene expression or gene regulation that is also highly correlated with the human trait originally assayed. In our study, we found an overall hypermethylation in bipolar subjects, as well as increased methylation associated with the well-established risk A-allele for susceptibility SNP rs1006737. Our study opens up the possibility that the non-coding *CACNA1C* risk SNPs may confer risk of disease through altered methylation in intron 3.

Altered DNA methylation within a gene, as observed in this study, has been linked to abnormal gene expression, alternative mRNA splicing and use of alternative promoters.^28, 29, 30, 31^ The exact relationship between DNA methylation and gene expression is, however, greatly dependent on the genomic location and tissue.^29^ It is therefore not straightforward to predict the effect and origin of the increased methylation found in blood of bipolar subjects in this study.

Previous studies investigating the correlation between *CACNA1C* risk genotypes and gene expression have found opposite effects of the risk allele depending on the investigated tissue. In human cerebellum, the risk A-allele of rs1006737 was correlated with decreased *CACNA1C* mRNA expression,^18, 32^ while the same allele was correlated with increased expression in human post-mortem dorsolateral prefrontal cortex^9^ and in induced human neurons.^33^ In the GTEx portal (V6 Release)^34^ a single significant eQTL for *CACNA1C* expression (rs7297582 in *CACNA1C* intron 3) was identified in cerebellum, with the minor allele correlating with reduced expression. As LD exist between rs7297582 and rs1006737 (*r*^2^=0.71), these data are in line with the previously published studies in cerebellum.^18, 32^ No shared eQTLs exist between blood and brain in GTEx with the current sample size (338 whole blood).

Whether the CGI 3 hypermethylation observed in our study was driven directly or indirectly by the nearby risk alleles is unknown. A recent study investigating the mechanistic relationship between the SNPs, DNA methylation and gene expression found that genetic variation can act either directly (SNP affects methylation, which change gene expression) or indirectly (SNP affects gene expression, which affects methylation level) on DNA methylation, and that the effect may vary with tissue.^35^ Studies addressing methylation and *CACNA1C* expression across different brain regions are necessary to fully uncover the molecular effect of increased *CACNA1C* methylation.

Increased *CACNA1C* CGI 3 methylation was detected in females. It is unclear if this gender-specific methylation effect is related to disease because this difference was also observed in the control subjects. Gender differences have previously been reported in *CACNA1C* genetic association studies, for example, Dao *et al.*,^36^ found female-only association between mood disorders and multiple intron 3 SNPs, that included rs1006737. Gender-specific association was also observed by Strohmaier *et al.*,^37^ who reported gender-specific impacts of rs1006737 in *CACNA1C* on a range of personality traits, resilience factors and depressive symptoms in 3793 healthy subjects. These studies differ in the rs1006737 alleles that were associated with mood disorders and their endophenotypes and so the impact of gender on genetic association at this locus remains unclear.^37^ It is possible that increased baseline DNA methylation of CGI 3 in females may provide a mechanism through which the risk allele of *CACNA1C* could determine sex-specific endophenotypes of BD.

A potential limitation of this study is the use of blood samples for methylation analysis. One argument for using blood samples for DNA methylation in psychiatric disorders is the common accessibility to such samples, providing adequate statistical power to detect small effects and mQTL. Moreover, recent studies have indicated that the DNA methylation status of many CpG sites in the brain are mirrored in the blood,^38^ and that common mQTL also exist between these two tissues, supporting the use of blood as a surrogate tissue to delineate disease-relevant processes within the brain.^39^

It is possible that medication influences DNA methylation status of *CACNA1C.* Two recent studies investigated the effect of commonly used mood stabilizers (including lithium, valproate and carbamazepine) on human methylome in neuroblastoma cells and whole blood of BD patients and reported significant drug-related changes in DNA methylation at several genes.^40, 41^ However, both of these studies used the Infinium HumanMethylation27 BeadChip array (Illumina, San Diego, CA, USA), where CGI 3 of *CACNA1C* is not covered, making a more comprehensive analysis of the effects of mood stabilizers on *CACNA1C* methylation of high interest in the future.

Overall, we have identified *CACNA1C* hypermethylation in whole blood of BD patients. Our study indicates that the regulatory effect of risk alleles in intron 3 is accompanied by an upwards shift in DNA methylation of the intron 3 CGI. In conclusion, *CACNA1C* DNA methylation may play a role in BD.

## Figures and Tables

**Table 1:** Overview of association between DNA methylation and BD and mQTL for CGI 3 of *CACNA1C*

	*Methylation difference between BD cases and controls* P*-value*			*Focused mQTL analysis*			*Full mQTL analysis*			*rs1006737 as mQTL*
*CpG site*	*All individuals*	*Females only*	*Males only*	*mQTL (most significant)*	P*-value*	P*-value (Bonferroni-corrected 24 markers)*	*mQTL (most significant)*	P*-value*	P*-value (Bonferroni-corrected 2634 markers)*	P*-value*
7	**6.16E−07**	**1.39E−04**	**9.06E−03**	rs2239030	**4.36E−05**	**1.05E−03**	rs2239030	**4.36E−05**	1.15E**−**01	**9.52E−03**
26	**7.15E−07**	**1.60E−04**	**1.23E−02**	rs2239030	**4.74E−04**	**1.14E−02**	rs4026015	**1.49E−05**	**3.92E−02**	**2.85E−02**
30	**2.00E−05**	**7.20E−03**	**7.17E−03**	rs2238056	**2.90E−07**	**6.96E−06**	rs2238056	**2.90E−07**	**7.64E−04**	**1.36E−04**
34	7.70E**−**01	3.54E**−**01	7.68E**−**01	rs2238056	**1.38E−04**	**3.32E−03**	rs2238056	**1.38E−04**	3.65E**−**01	6.73E**−**02
35	**1.16E−07**	**1.01E−03**	**6.41E−04**	rs2238056	**2.63E−07**	**6.32E−06**	rs2238056	**2.63E−07**	**6.94E−04**	**3.02E−04**
42	**8.66E−07**	**1.54E−04**	**3.09E−02**	rs10848634	**2.14E−07**	**5.13E−06**	rs10848634	**2.14E−07**	**5.63E−04**	**8.47E−05**

Abbreviations: BD, bipolar disorder; CGI, CpG island; mQTL, methylation quantitative trait loci.

Entries marked in bold indicate findings with *P*-value <0.05.
