# Supplementary figures and images for: CACNA1C hypermethylation is associated with bipolar disorder
Source: Transl Psychiatry. 2016 Jun 7;6(6):e831–. doi: 10.1038/tp.2016.99 (PMC4931616; doi:10.1038/tp.2016.99)

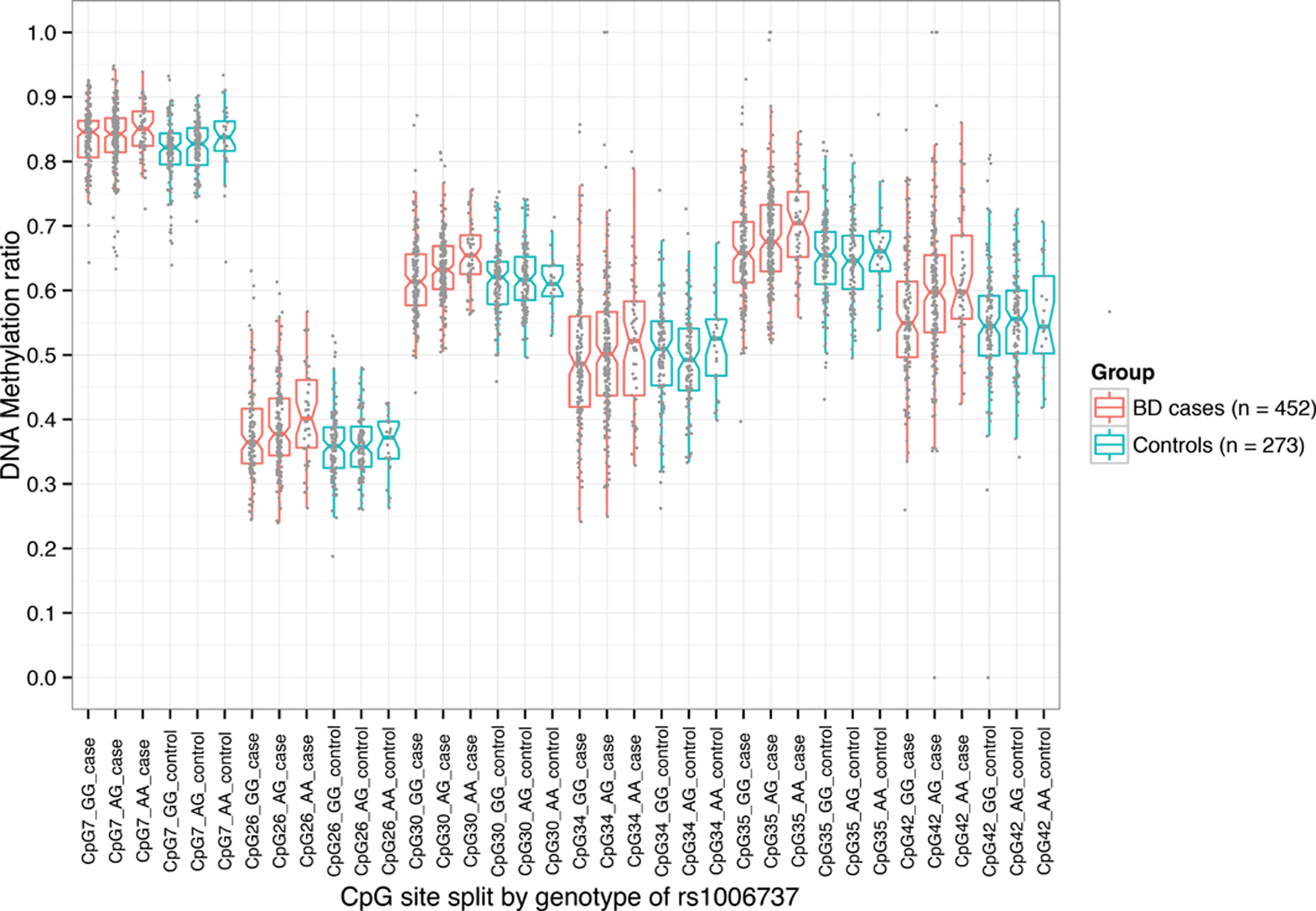

Supplement: Supplementary Figure 1 [file tp201699x1.tif]
