# Supplementary material for: CACNA1C hypermethylation is associated with bipolar disorder
Source: Transl Psychiatry. 2016 Jun 7;6(6):e831–. doi: 10.1038/tp.2016.99 (PMC4931616; doi:10.1038/tp.2016.99)
Supplement: Supplementary Information [file tp201699x2.docx]

**Supplementary Tables**

**Supplementary Table 1.** Overview of samples used for methylation analysis

| **Samples** | **BD cases** | **Control** | **All individuals** |
| --- | --- | --- | --- |
| Methylation | 582 | 319 | 901 |
| Genotypes | 452 | 273 | 725 |
| Males/Females | 225/357 | 159/160 | 901 |
| Bipolar I/Bipolar II | 542/30 | - | 572 |
| Family history of psychiatric disease (maternal/paternal/none) | 185/142/111 | - | 438 |

**Supplementary Table 2.** EpiTYPER primers used for methylation profiling of specific CpG sites across five CpG islands in *CACNA1C*

| Targeted region | Primer name | Primer sequence (5’ – 3’) |
| --- | --- | --- |
| CGI 1 | CACNA1C_1_01_L | aggaagagagTTGTTTTTGTAGAAATAGTTTTTGTTAGA |
|  | CACNA1C_1_01_R | cagtaatacgactcactatagggagaaggctAATCCTCCTAAAACCCCTACCC |
|  | CACNA1C_1_02_L | aggaagagagGGGTAGGGGTTTTAGGAGGATT |
|  | CACNA1C_1_02_R | cagtaatacgactcactatagggagaaggctCCAACCTTACCTTAATAATTTTCCTCT |
|  | CACNA1C_1_03_L | aggaagagagAGGAAAATTATTAAGGTAAGGTTGGA |
|  | CACNA1C_1_03_R | cagtaatacgactcactatagggagaaggctACAACCACTTAAAAAACTTTTCCCC |
|  | CACNA1C_1_04_L | aggaagagagGGGGAAAAGTTTTTTAAGTGGTTG |
|  | CACNA1C_1_04_R | cagtaatacgactcactatagggagaaggctCCATCCTCAAATACTAACCCACAT |
| CGI 2 | CACNA1C_2_01_L | aggaagagagTTGTTTATTTAAGGTGAGGTAAGGAGA |
|  | CACNA1C_2_01_R | cagtaatacgactcactatagggagaaggctAAAAATATACTCAAAAACCAACCCC |
|  | CACNA1C_2_02_L | aggaagagagGGGGTTGGTTTTTGAGTATATTTTT |
|  | CACNA1C_2_02_R | cagtaatacgactcactatagggagaaggctTAACCCAACACAACTAACACAACTC |
|  | CACNA1C_2_03_L | aggaagagagTGGGAAATTTAAGAAGTAGGGTAGT |
|  | CACNA1C_2_03_R | cagtaatacgactcactatagggagaaggctCCAACCCAAACAACTTATTATACAAAC |
| CGI 3 | CACNA1C_3_01_L | aggaagagagGGAAGGAGGTTAGAGTTATTGTAGATTT |
|  | CACNA1C_3_01_R | cagtaatacgactcactatagggagaaggctCCATTACTCCTCACAAAACACCTTA |
|  | CACNA1C_3_02_L | aggaagagagTAAGGTGTTTTGTGAGGAGTAATGG |
|  | CACNA1C_3_02_R | cagtaatacgactcactatagggagaaggctAACACTCAAATTCTACCCTCAACTTT |
| CGI 4 | CACNA1C_4_01_L | aggaagagagGAGGGGAAAAAGATTATTATGTAGGA |
|  | CACNA1C_4_01_R | cagtaatacgactcactatagggagaaggctAACCCTACTAAAAACCAAAAACACC |
|  | CACNA1C_4_02_L | aggaagagagTTAGTTTTTAGGGGTTTTATTGGAAA |
|  | CACNA1C_4_02_R | cagtaatacgactcactatagggagaaggctCTCCACAACCAAATCAAAAAAATAC |
|  | CACNA1C_4_03_L | aggaagagagGGGTGTTAAGGGGTTTTTTTATTTA |
|  | CACNA1C_4_03_R | cagtaatacgactcactatagggagaaggctTAACCAACCTCCCACTTACCTATTA |
|  | CACNA1C_4_04_L | aggaagagagTTAATAGGTAAGTGGGAGGTTGGTT |
|  | CACNA1C_4_04_R | cagtaatacgactcactatagggagaaggctAATCACATTCTACCACTCAAAAAAT |
| CGI 5 | CACNA1C_5_02_L | aggaagagagAGGATTGGGGTAGTTTGTTTAAGAT |
|  | CACNA1C_5_02_R | cagtaatacgactcactatagggagaaggctAATCCCAACAAAAATAAATAAAAAC |
|  | CACNA1C_5_03_L | aggaagagagGTTTTTATTTATTTTTGTTGGGATTAGA |
|  | CACNA1C_5_03_R | cagtaatacgactcactatagggagaaggctTAAAATCCTCCTAAAAATCCTACCC |
|  | CACNA1C_5_04_L | aggaagagagGGGTAGGATTTTTAGGAGGATTTTA |
|  | CACNA1C_5_04_R | cagtaatacgactcactatagggagaaggctAATATCAAACCACAAAACAAAAAAA |
|  | CACNA1C_5_05_L | aggaagagagTTTTTTTGTTTTGTGGTTTGATATT |
|  | CACNA1C_5_05_R | cagtaatacgactcactatagggagaaggctCAAATACATTACCAATTACCTTTCCA |
|  |  |  |

L,forward; R,reverse; CGI, CpG island

**Supplementary Table 3.** iPLEX primers used for methylation analysis of six CpG sites in the CpG island (CGI 3) in *CACNA1C* intron 3

| Primer | Primer sequence (5’ – 3’) |
| --- | --- |
| CpG7_T15_EXT | CCICTTCTTCCCCTTCC |
| CpG7_T15_F | ACGTTGGATGAAAACCACACCTCTCCAA |
| CpG7_T15_R | ACGTTGGATGGAGGGGTTTGTTTTGTGTG |
| CpG26_A-13_EXT | gggcAGGGIGTTATGGGTGTA |
| CpG26_A-13_F | ACGTTGGATGTAGGTAGTGAGGGGTTGTTG |
| CpG26_A-13_R | ACGTTGGATGTACCCTCAACAAACCCTAAC |
| CpG30_T9_EXT | ACCCTAACIACTCAACC |
| CpG30_T9_F | ACGTTGGATGCCTCAACAAACCCTAACIAC |
| CpG30_T9_R | ACGTTGGATGTAGGTAGTGAGGGGTTGTTG |
| CpG34_A-11_EXT | GGGTTTTTIGTTTTGGTGG |
| CpG34_A-11_F | ACGTTGGATGTAGGGTTTGTTGAGGGTAAG |
| CpG34_A-11_R | ACGTTGGATGCCATTACTCCTCACAAAACAC |
| CpG35_EXT | TCCTCACAAAACACCTTAC |
| CpG35_F | ACGTTGGATGCCATTACTCCTCACAAAAC |
| CpG35_R | ACGTTGGATGTAGGGTTTGTTGAGGGTAAG |
| CpG42_EXT | TTCTCAATCTCTTCCTAAAAAATC |
| CpG42_F | ACGTTGGATGCACTCAAATTCTACCCTCAAC |
| CpG42_R | ACGTTGGATGGAGTTTGGAAGTATAGTTAG |

F, forward; R, reverse; EXT, extension; I, inosine

**Supplementary Table 4.** Spearman’s correlation of DNA methylation levels between different CpG sites in CGI 3 of *CACNA1C*. Rho indicates the rank correlation

|  | **CpG7** | | **CpG26** | | **CpG30** | | **CpG34** | | **CpG35** | | **CpG42** | |
| --- | --- | --- | --- | --- | --- | --- | --- | --- | --- | --- | --- | --- |
|  | rho | p-value | rho | p-value | rho | p-value | rho | p-value | rho | p-value | rho | p-value |
| **CpG7** | 0.83 | 8.30E-01 | 0.31 | 1.81E-17 | 0.40 | 6.33E-36 | 0.32 | 4.94E-22 | 0.46 | 7.05E-48 | 0.45 | 2.78E-42 |
| **CpG26** |  |  | 0.37 | 3.76E-01 | 0.43 | 2.26E-33 | 0.18 | 1.67E-06 | 0.43 | 4.99E-34 | 0.51 | 1.40E-48 |
| **CpG30** |  |  |  |  | 0.62 | 6.25E-01 | 0.66 | 1.72E-110 | 0.84 | 3.21E-241 | 0.83 | 7.85E-213 |
| **CpG34** |  |  |  |  |  |  | 0.50 | 5.00E-01 | 0.69 | 7.82E-128 | 0.65 | 4.13E-104 |
| **CpG35** |  |  |  |  |  |  |  |  | 0.66 | 6.66E-01 | 0.82 | 4.12E-207 |
| **CpG42** |  |  |  |  |  |  |  |  |  |  | 0.57 | 5.66E-01 |

**Supplementary Table 5.** Genetic association analysis of the top mQTLs for CGI 3 in the UCL sample and PGC-BP ^7^ (accessed through Metamodics ^26^)

|  | **Association analysis in UCL sample** | | | | | | | **mQTL in UCL sample** | **Association analysis in BD PGC** | | | | |
| --- | --- | --- | --- | --- | --- | --- | --- | --- | --- | --- | --- | --- | --- |
| **SNP** | **A1** | **F_A** | **F_U** | **A2** | **P -value** | **OR** | **SE** | **Top mQTL for CpG** | **A1** | **A2** | **P-value** | **OR** | **SE** |
| **rs1006737** | A | 0.3719 | 0.3238 | G | 0.03 | 1.24 | 0.095 | - | A | G | 0.000017 | 1.11 | 0.024 |
| **rs2239030** | A | 0.4755 | 0.4323 | G | 0.05 | 1.19 | 0.091 | CpG7 (0.00004356), CpG26 (0.0004743) | A | G | 0.0077 | 1.06 | 0.023 |
| **rs2238056** | C | 0.5031 | 0.4697 | T | 0.14 | 1.14 | 0.090 | CpG30 (2.902E-07), CpG34 (0.0001384), CpG35 (2.633E-07) | C | T | 0.0022 | 1.07 | 0.023 |
| **rs10848634** | C | 0.4329 | 0.3882 | T | 0.05 | 1.20 | 0.092 | CpG42 (2.138E-07) | C | T | 0.0012 | 1.08 | 0.024 |

**Supplementary Figure 1**. DNA methylation levels at six CpG sites in CpG island 3 of *CACNA1C* split by genotype of rs1006737
